# Functional neurological disorder: lighting the way to a new paradigm for medicine

**DOI:** 10.1093/brain/awab358

**Published:** 2021-10-04

**Authors:** Mark J Edwards

**Affiliations:** Institute of Molecular and Clinical Sciences, St George’s University of London, London SW17 0QT, UK

## Abstract

In this Essay, Mark Edwards argues that the plight of people with functional neurological disorder within healthcare highlights a general problem with a broken paradigm of modern medicine. He argues that the passivity of the traditional sick role needs replacing with a participatory, rehabilitative medical practice.

***What if the patients most health professionals actively seek to avoid, people with ‘medically unexplained’ or functional symptoms, were those who hold the key to a more successful, more rewarding and more just system of medical practice for all? I think they do. They force us to answer the question, to paraphrase Wittgenstein: What is left over, if I subtract the fact that I have a disease, from the fact that I am ill? Within the answer to this question is the human, participatory aspect of illness, which, despite hundreds of mission statements to the contrary from healthcare organizations the world over, is not adequately addressed in our medical training, practice and principles. We can and should do better, and this is a proposal for how***.

In his 1971 book *A Theory of Justice*,^1^ American philosopher John Rawls suggests an intriguing thought experiment. Imagine that a group of people sat down together to devise the basic rules by which society should be organized. But, instead of knowing their circumstances, they met behind a ‘veil of ignorance’. For Rawls this meant that although those people trying to devise the rules knew the different opportunities, challenges and inequalities that exist in society, they had no knowledge of their own personal situation. So, what decisions would a rational person make about societal rules and organization if they did not know their lot in the lottery of life? In devising this scenario, Rawls cleverly pulls our inherent urges for self-protection and preservation into a societal, outward looking focus. In doing so, these selfish characteristics are hijacked in the cause of fairness for all.

There have been some criticisms of this approach and indeed revision of the idea by Rawls himself, but it remains, I think, an interesting experiment. It acknowledges the randomness of the distribution of opportunities and attributes to human beings and seeks despite this to find a kind of natural justice.

A certain kind of natural justice is also evident when it comes to societal attitudes to and treatment of people who are ill. Most commonly bundled together in the concept of the ‘sick role’, illness allows the shedding of certain personal and societal responsibilities, and the receipt of certain personal and societal rewards. The linguistic inference is an interesting one here: the person who is sick needs to play their part, and if they do so correctly, the other actors will play their parts too.

So, what is the casting director looking for in the ideal actor for the sick role? I would argue that in modern medical practice, the ideal actor is one for whom the illness has maximum separation from the self.

A doctor’s surgery. Seated is doctor, surrounded by medical paraphernalia. Enter stage left, sick person, carrying before them their diseased body.

sick person I seek help! I have received the following symptoms from my body.

[She hands a list to doctor.]

I present them to you in the order in which they arrived, taking care to remove any personal or sociocultural bias from their description and adopting behaviour and language as specified in the How to be a Good Patient Handbook Volume 5, paying particular attention to Chapter 17: Making a Fuss Over Nothing and How to Avoid it.

doctor Thank you. Your work here is done. Let me relieve you of that burden.

[He takes the body.]

I’ll have a look at this and get it back to you when I can.

Conversely, the villain of our medical drama, our ultimate ‘bad actor’, is a discomforting individual whose supposed illness is mixed up so irreconcilably with personal behaviour that it is not possible to see where one stops and the other begins.

## The collision between the sick role and the nature of neurological and psychiatric illness 

The sick role is therefore one that embodies a fundamental *passivity*. This passivity is essential because the greater it is, the less my predicament is about me, my responsibility, my fault. Given this, it makes sense that when dysfunction occurs in faculties that we most closely associate with personal will and control—movement, thoughts, feelings, those things that are ultimately the building blocks of behaviour—the actors are viewed with most suspicion. ‘People keep thinking I’m drunk’ says the man with cerebellar ataxia. ‘People keep staring at me like I’m going to kill them!’ says the woman who shouts out at the voices she hears commenting on her every action. The stigma of neurological and psychiatric illness is one that at least in part relates to a perceived violation of the blamelessness of the sick role.

One approach to resolve this stigma has been to locate such illnesses firmly in the biology of the brain. I’m not behaving badly, my brain is! This neuro-centric approach appears to have a logic behind it. Given that the brain is an essential requirement for there to be behaviour and mental experience in the first place, dysfunction in behaviour and mental experience must be created by and encoded within the brain. An alternative approach, championed episodically within and sometimes against psychiatry, is to locate such illnesses firmly within society. It is therefore the external forces of parenting, societal hierarchies, cultural expectations, and many others from which you might wish to take your pick, which conspire against the individual to determine mental dysfunction and distress.

These dichotomies are played out across medical practice and research. The clearer the biological correlate of the illness, the more neuro-centric is the approach. The less clear the biology, the more psychosocial the approach. Brain versus mind, organic versus non-organic, bio versus psychosocial: an unwinnable Health World Cup. But bizarrely, whatever side one is on, the outcome of these splits for the person who is ill is similar. Whether it is ‘all in the genes’, or ‘all in the sociocultural milieu’ the result is passivity. It seems that by trying to distil the biological or sociogenic illness from the person, done for their own protection from stigma, we end up discarding the most important bit of all. That it feels like something, personally, to be ill. The junction of biology and society is within me, and it cannot exist without me. The facts of molecules, neural signals, family and social dynamics are given life within me. *And this is not a passive process*. As with Schrodinger’s infamous cat, the act of measurement, of bringing to life, of actualization, is an act of creation and therefore of change. There is participation here that cannot be removed, and nor should we seek to, as this participation is the essence of existing as a conscious human being.

## Functional neurological disorder: a human red rag to the twin bulls of biomedicine and psychosocial medicine

The healthcare journeys of people with functional neurological disorder shine a harsh spotlight on the unintended consequences of medical practice founded on passivity rather than participation. The enormously expensive but largely anti-therapeutic activity within healthcare related to people with functional symptoms^2-5^ appears to me to be mainly driven by an unsuccessful application of current models of illness and treatment. Such patients present to doctors with physical symptoms and signs, which indicate the presence of disease, but when we drill down into the body, no disease is found. More worryingly, the symptoms and signs break fundamental rules of disease and damage: they fluctuate with attentional state, they flit from one system to another, they align themselves with personal and societal beliefs about how disease might manifest and not with basic physical and biological laws. But worse is to come.

When we seek, with the best of intentions and using the only tools we have left, to reinterpret the symptoms as manifestations of psychosocial adversity, we are left with an explanatory gap at both a conceptual and personal level. Conceptually, how and why has psychosocial adversity, which is a general risk factor for so many illnesses and may have occurred years before, led in this person at this time to the development of these particular symptoms? And personally, if my symptoms affect my body and not my mind, how can they legitimately have been caused by psychosocial adversity, even if this exists in my life story, which it may not? Without the fig leaf of the sick role from either side, what is left? Only the personal, and with it the responsibility for my behaviour. I am fundamentally ‘not doctorable’.

This non-doctorability lies at the heart of the conflictual relationship between people with functional symptoms and healthcare professionals and organizations. The existential limbo inhabited by those with functional symptoms was neatly encapsulated by a recent patient of mine who recounted her experience of being told by an earnest young doctor that her symptoms were: ‘very real … for you’. The personal experiences of patients and of healthcare professionals who treat them reveal distress and anger on both sides. There are very high levels of ‘demand failure’, where people present repeatedly to diagnostic and treatment services which cannot meet their needs. The vacuum in adequate understanding and treatment leaves the field open for both well-meaning and unscrupulous purveyors of pseudoscientific explanations and treatments. The human, empathic response of some talented clinicians seeing this disastrous level of care and lack of compassion for people who are so clearly ill can sometimes become completely misdirected. This often manifests as a crusade based around a single biological explanation for symptoms such as a specific infection or type of inflammation, apparently saving people from medical limbo, but instead simply moving them to another part of a fundamentally broken model of medicine.

## The iceberg beneath

It may be tempting to just turn aside from the predicament of people with functional neurological disorder, safe perhaps in the knowledge that even though our systems may not be perfect for them, we are at least on the right track when it comes to helping people who are ill with a disease. However, I would argue that there is no safe haven to be found here either. If our mission to provide excellence in healthcare has been proven to be more ‘Mission Impossible’ than ‘Mission Accomplished’ for people with functional neurological disorder, then this will also be the case for other people who are ill. Because, returning to the theme above, being ill with anything is personal.

While my diseased organ can be separately analysed and quantified and the socio-cultural context of my existence can be measured and defined, it is within me that they are brought to life. As with any living creation, this act of conscious experience fundamentally changes the component parts. And what if the organ which is most relevant in mediating this transformation is also the organ that is damaged, deranged or diseased? Then it is likely that the personal complexity of my illness will be even greater. Therefore, people whose brains are made different by influences of development, disease, damage, environment and experience will be most likely to seek help for complex problems that cannot be solved within our current models of healthcare. And nor is this just a problem for neurology and psychiatry services. From people with heart disease to those with chronic lung disease, biological measures of disease correlate extremely poorly with measures of disability, quality of life and distress.^6^^,^^7^

We are perplexed by this and may even label it a paradox.^8^ We are tempted to look at our measuring devices for the answers—maybe we just need to improve our scans, blood tests and disability questionnaires? But the real answer is obvious: *consciousness breathes life into pathology*, as it does to the physical and social environment. This process, mediated by individual bodies and brains, gives birth to a feeling, an experience, occurring in a place and time and which is in turn changed and given new life through interaction with others who are conscious. The true paradox is that we continue to act as though a relentless and single-minded focus on improving the tools we have for measuring disease and altering the associated pathophysiological state of the body will solve all the problems of people who are ill.

## Closing the epistemic gap: from participation to rehabilitation

The first step in solving this dilemma is to close the epistemic gap in our concept of what it is to be ill. Being ill, as opposed to having a disease, requires a being, and therefore can only exist as a participatory state. The illness is brought to life in the person, and their participation is therefore a fundamental prerequisite for it to exist. Our current concepts of illness seek to avoid this fact because if we admit to participation, then we imply personal responsibility residing in those who are ill. However, this is only problematic if we view such personal responsibility in a Cartesian fashion as something that is fundamentally and categorically different from other aspects of illness and therefore untouchable medically and scientifically.

Instead, if we view this participatory aspect as the actual foundation of being ill, then there is only one logical mode of operation for healthcare, and this mode is *rehabilitation*. I use this word to encapsulate an ideal of participatory medicine. Rehabilitation cannot be done to people—it requires their participation. A rehabilitatory mode of operation forces the personal to the front of all interactions and decisions. The participation and responsibility of the person who is ill is not treated as a separate entity, divorced from the process of healthcare, but is instead an essential and legitimate focus of assessment and treatment too. Just as consciousness breathes life into pathology and environment, rehabilitation breathes life into medicine. The ideal of rehabilitation is like a positive to the negative of illness, the perfect mirror, matching every edge and surface, able to sense and influence every aspect.

## From ideals to mission statements and reality

I anticipate that some of you might be feeling a sense of tedious inevitability triggered by these arguments. Perhaps you are picturing a field of straw men being created, to enable a pseudo-heroic destruction of a thing that was never there in the first place. Because, if behind all the quantum neurobabble above, all I am really saying is that we need to put the person back into medicine, haven’t we done that already? After all it has been 45 years since Engel wrote about the bio-psycho-social model of illness and the need to replace the narrow biomedical model.^9^ Hasn’t this battle been fought and won years ago? Just look at a mission statement from any healthcare organization and you won’t be able to avoid tripping over references to ‘whole person medicine’, ‘bio-psycho-social approach’, ‘person-centred care’ and the like. The extreme position of the anti-psychiatrists such as Thomas Szasz has been modulated, assimilated, and in a filtered form has appropriately influenced the care of those with mental illness. We are so much more enlightened now. Look at all the articles in the popular press about ‘mental health issues’. No health without mental health! What more do you want?

However, our failures to make any significant inroads into poor long-term outcomes for people with functional neurological disorder or to close the ‘paradoxical’ gap between disease metrics and disability for those with other illnesses, tell a different story. This is a story where, as is so often the case, the PR of mission statements does not reflect reality. Nearly 60 years ago, Denis Hill wrote of how neuropsychiatry could be the bridge joining the biological focus of neurology with the personal focus of psychiatry for the benefit of all those with disturbance of mental and neurological function.^10^ Rather than stoke the antagonism between biological psychiatry and psychoanalysis, Hill argued that in some form both were necessary, and neither was sufficient.

Studies revealing the prevalence and impact of psychiatric disorders in those with neurological disease such as epilepsy and Parkinson’s disease have clearly shown the importance of merging psychiatric expertise in diagnosis and treatment with neurological expertise. However, despite this, neuropsychiatry and counterparts such as behavioural neurology have remained small super-specialities. Further bridges have been constructed that focus on the personal experience and consequences of illness, often encompassing people with functional neurological symptoms. In Germany and elsewhere, there are traditions of psychosomatic medicine where people can access a certain style of specialist assessment and rehabilitation. Health Psychology departments offer therapy to assist people with the psychological impact of being ill and the overlap between anxiety, functional somatic symptoms and other illness. Neurorehabilitation services will often have neuropsychologists within their teams, providing expertise in rehabilitation of cognition and behaviour for people with brain damage and disease, such as traumatic brain injury and encephalitis.

However, these bridges, constructed with care and expertise by talented and visionary clinicians, have not been successful in solving our dilemma. In my view this is precisely because they are constructed as bridges, attempting to solve the problem by joining something on to the rest of medical practice. This means that thing that is connected remains ‘over there’, an add-on, a nice, but optional extra and not the essential, core business of medicine. Thus, the incredible expertise, passion and commitment that exists within rehabilitation services is somewhere else, situated over a bridge which people must travel across after the ‘real’ biomedical work is done, always an easy target when costs need to be cut or more space needs to be found for expansion of another department.

Rehabilitation services themselves, as currently constructed, face a number of structural challenges. They are often split along mind/body lines, compartmentalizing expertise and compromising care for all patients regardless of their diagnosis, failing to learn the lesson that having an ‘organic’ disease does not provide immunity from also having functional symptoms or psychiatric illness and vice versa: in fact co-occurrence is almost inevitable. The multidisciplinary nature of rehabilitation highlights potentially toxic and unresolved splits in medicine, for example doctors versus allied health professionals, psychiatrists versus psychologists, physical rehabilitation versus cognitive and behavioural rehabilitation. The outcome of these structural issues in rehabilitation is a tendency for fragmentation, like a jigsaw constructed again and again but always ending up with a missing piece. By splitting or siloing rehabilitation services we set ourselves up to fail precisely those people who have the greatest need of our help. If our aim is to help with biopsychosocial, whole person complexity, then we need to model this within our services, rather than to pretend that creating a compound word out of specialisms or putting the word ‘multidisciplinary’ in front of our team name automatically makes us fit for purpose.

There is another challenge for rehabilitation that is perhaps best summed up by the word 'legitimacy'. If we believe (quite rightly) that the randomized controlled trial has been a great tool to find effective pharmacological treatments, how do we deal with the problem that, as currently constituted, these methods are not easily applicable to understanding the complex interface between biology and environment that occurs within humans who are ill, and therefore to studying the process of rehabilitation? Without the protection of the scientific method, rehabilitation becomes an easy target for accusations of medical illegitimacy, and for the rise of guru-ism and dogma. And, if whatever assessment or treatment that is done is not easily amenable to validation in the evidence-based way we have come to expect, then is it really any different from nice people being nice to other people who are ill? So, it seems entirely appropriate to replace the hard, powerful words we use for proper medicine such as ‘intervention’ or ‘treatment’ with softer words such as ‘support’ or ‘care’. And surely anyone can offer care and support, so it should be cheap. A bit of talking, a bit of movement—that’s easy, no expertise needed here! These problems and more directly delegitimize the professionals and services who work at the biopsychosocial interface within rehabilitation, and by implication, the people that they treat.

## Rehabilitation as the purpose of medicine

To move forward, we first need to recognize that the crown of legitimacy claimed by evidence-based medicine is tarnished, precisely because it fails to deal with the influence of the personal in medicine. The extent to which a treatment will change the experience of being ill is not easily predictable from the published results of clinical trials. This does not mean we should reject the method. However, if we really want to see the benefits of the scientific revolution in medicine, we need to solve the hard, and fundamentally scientific problem of developing and testing treatments that improve illness as well as disease.

The solution is not to claim, as some in the psycho-analytical tradition have, that what happens in such treatment is so different, so fundamentally ineffable, that it cannot be subject to the harsh lens of scientific evidence. The opposite is true. Understanding the mechanisms of interaction between disease and illness, devising methods to treat both in parallel and finding ways to implement such practice widely within healthcare are the places where the precision and ruthlessness of the scientific method meet their hardest, most complex, but most vital challenge. We can meet this challenge, but only if we recognize that *the actual purpose of medical practice is rehabilitation*. A rehabilitative mode of operation is medicine and so by default has to be present within every medical encounter and service. If a person wishes to characterize themselves as a clinician or a clinician-scientist, then this rehabilitative mode of operation has to be the foundation of their clinical practice and research. It cannot just be outsourced to someone else ‘over there’. If we commit to this ideal and mission, then we will find a way to innovate and deliver it.

Returning to the Rawlsian thought experiment, it is my belief that a group of rational individuals, not knowing their biopsychosocial circumstances, would make a priority of researching and delivering healthcare that is centred on the personal, as the place where the biological and environmental meet and are given life. This means that a just healthcare system should have as its basic foundation an interdisciplinary, rehabilitative mode of operation, that allows different levels of description, different aspects of expertise and different perspectives to pervade all those who are participating, including the person who is ill. There are many challenges to delivering this, and no easy solutions. The aim is not to create a cuddly version of biomedicine, a rebranding exercise where we expect change to happen by installing a few aromatherapy diffusers and inserting the word ‘wellness’ into our mission statements. Instead, it is to recognize and invest in the science and expertise that will truly allow us, as scientists and clinicians, to become partners with the patient. Partners in understanding, moulding and finally recreating in a better form what it is, personally, to be ill.
